# Streptozotocin and Alloxan-based Selection Improves Toxin Resistance of Insulin-Producing RINm Cells

**DOI:** 10.1155/EDR.2000.211

**Published:** 2000

**Authors:** Konstantin O. Bloch, Romy Zemel, Olga V. Bloch, Hagar Grief, Pnina Vardi

**Affiliations:** 1 Diabetes and Obesity Research Laboratory Felsenstein Medical Research Center of Tel-Aviv University Petah Tikva Israel; 2 Molecular Hepatology Laboratory Rabin Medical Center and Felsenstein Medical Research Center of Tel-Aviv University Petah Tikva Israel; 3 Gino Stock Dermatophysiology Laboratory Dana Children Hospital Sourasky Medical Center Tel-Aviv Israel

## Abstract

The aim of our study was to develop a method for selection of subpopulations of insulin producing RINm cells with higher resistance to beta cell toxins. Cells, resistant to streptozotocin (RINmS) and alloxan (RINmA), were obtained by repeated exposure of parental RINm cells to these two toxins, while the defense capacity, was estimated by the MTT colorimetric method, and [^3^H]-thymidine incorporation assay. We found that RINmS and RINmA displayed higher resistance to both streptozotocin (STZ) and alloxan (AL) when compared to the parental RINm cells. In contrast, no differences in sensitivity to hydrogen peroxide were found between toxin selected and parental cells. Partial protection from the toxic effect of STZ and AL was obtained only in the parental RINm cells after preincubation of cells with the unmetabolizable 3- O-methyl-glucose. The possibility that GLUT-2 is involved in cell sensitivity to toxins was confirmed by Western blot analysis, which showed higher expression of GLUT-2 in parental RINm compared to RINmS and RINmA cells. In addition to the higher cell defense property evidenced in the selected cells, we also found higher insulin content and insulin secretion in both RINmS and RINmA cells when compared to the parental RINm cells. In conclusion, STZ and AL treatment can be used for selection of cell sub-populations with higher cell defense properties and hormone production. The different GLUT-2 expression in parental and re sistant cells suggest involvement of GLUT-2 in mechanisms of cell response to different toxins.


*Int. Jnl. Experimental Diab. Res.*, Vol. 1, pp. 211–219
Reprints available directly from the publisher
Photocopying permitted by license only

© 2000 OPA (Overseas Publishers Association) N.V.
Published by license under
the Harwood Academic Publishers imprint,
part of The Gordon and Breach Publishing Group.
Printed in Malaysia.

### Streptozotocin and Alloxan-based Selection Improves Toxin Resistance of Insulin-producing RINm Cells

KONSTANTIN O. BLOCH[a,*], ROMY ZEMEL[b], OLGA V. BLOCH[c], HAGAR GRIEF[b] and PNINA VARDI[a]

[a]*Diabetes and Obesity Research Laboratory, Felsenstein Medical Research Center of Tel-Aviv University, Petah Tikva, Israel;* [b]*Molecular Hepatology Laboratory, Rabin Medical Center and Felsenstein Medical Research Center of Tel-Aviv University, Petah Tikva, Israel;* [c]*Gino Stock Dermatophysiology Laboratory, Dana Children Hospital, Sourasky Medical Center, Tel-Aviv, Israel*

*(Received in final form 22 December 1999)*

**The aim of our study was to develop a method for selection of subpopulations of insulin producing RINm cells with higher resistance to beta cell toxins. Cells, resistant to streptozotocin (RINmS) and alloxan (RINmA), were obtained by repeated exposure of parental RINm cells to these two toxins, while the defense capacity was estimated by the MTT colorimetric method, and [$^3$H]-thymidine incorporation assay. We found that RINmS and RINmA displayed higher resistance to both streptozotocin (STZ) and alloxan (AL) when compared to the parental RINm cells. In contrast, no differences in sensitivity to hydrogen peroxide were found between toxin selected and parental cells. Partial protection from the toxic effect of STZ and AL was obtained only in the parental RINm cells after preincubation of cells with the unmetabolizable 3-O-methyl-glucose. The possibility that GLUT-2 is involved in cell sensitivity to toxins was confirmed by Western blot analysis, which showed higher expression of GLUT-2 in parental RINm compared to RINmS and RINmA cells. In addition to the higher cell defense property evidenced in the selected cells, we also found higher insulin content and insulin secretion in both RINmS and RINmA cells when compared to the parental RINm cells. In conclusion, STZ and AL treatment can be used for selection of cell sub-populations with higher cell defense properties and hormone production. The different GLUT-2 expression in parental and resistant cells suggest involvement of GLUT-2 in mechanisms of cell response to different toxins.**

*Keywords*: Streptozotocin; Alloxan; RIN cells; 3-O-methyl glucose; GLUT-2; Insulin

*Abbreviations*: STZ, streptozotocin; AL, alloxan; RINmS, streptozotocin-selected RINm cells; RINmA, alloxan-selected RINm cells; 3-OMG, 3-O-methyl glucose; MTT, C,N-diphenyl-N′-4,5-dimethyl thiazol 2 yl tetrazolium bromide; GLUT-2, glucose transporter 2; FCS, fetal calf serum

### INTRODUCTION

Beta-cell lines constitute a potential source of genetically engineered insulin-producing cells for replacement of difficult-to-obtain human tissue for pancreatic islet transplantation.[1–3] Beta-cells are known to be susceptible to destruction, primarily by toxins, autoimmune

*Corresponding author. Diabetes and Obesity Research Laboratory, Felsenstein Medical Research Center, Tel-Aviv University, Rabin Medical Campus, Petah Tikva 49202, Israel. Tel.: 972-3-9376280, Fax: 972-3-9211478, e-mail: pvardi@post.tau.ac.il mechanisms, and by infectious agents, with several features of the destructive process common to all etiological factors.[4]

Alloxan (AL) was the first cytotoxic compound reported to cause inhibition of glucose-induced insulin secretion and selective beta-cell damage,[5] but insulin deficiency can also be induced by streptozotocin (STZ), which is a glucose analog (glucopyranose, 2-deoxy-2-[3-methyl-e-nitrosourido-D]) causing specific beta-cell destruction.[6] Both compounds cause fragmentation of nuclear beta-cell DNA, poly (ADP-ribose) polymerase activation, and NAD+ depletion.[4] The cytotoxic activity of these compounds seems to be achieved through their penetration into the beta-cells, a phenomenon which by itself depends on the expression of the glucose transporter protein-2 (GLUT-2).[7,8] An additional contributor to beta cell sensitivity to various toxins related to their poor antioxidant enzyme defense system.[9]

Recently, STZ was found to induce cell differentiation in pancreatic islets. Following exposure of cells to STZ, PDX-1 positive beta-stem cells differentiate into insulin-producing cells.[10] Beta-cell sensitivity to various toxins is not homogeneous, neither in pancreatic islets nor in tumor derived lines of insulin producing cells. Major differences exist also between human and rodent tissues, with rodents islet cells being more sensitive to diabetogenic toxins.[11,12] Beta-cell heterogeneity has also been found at the level of glucose sensitivity and intracellular insulin content.[13]

The RINm cell line which we used in the present study, was established from a transplantable, radiation-induced rat islet cell tumor,[3] a cell line commonly used by many investigators to study beta cell characteristics and function. In the present study, we report on a method for selection of toxin resistant cells, based on repeated exposure of parental RINm cells to a high dose of STZ and AL. This method allows for selection of cells with improved defense capacity and higher hormone production.

### MATERIALS AND METHODS

### Materials

Streptozotocin, alloxan, 3-O-methyl glucose (3-OMG), C,N-diphenyl-N′-4,5-dimethyl thiazol-2-yl tetrazolium bromide (MTT), bovine serum albumin and colchicine were purchased from Sigma (St. Louis, Mo., USA) and [$^3$H] thymidine (2 Ci/mmol) was purchased from ICN Pharmaceutical, Inc. (Costa Mesa, Ca., USA). The RPMI 1640 culture medium, fetal calf serum (FCS), penicillin, streptomycin, trypsin-EDTA solution and other reagents were obtained from Biological Industries Beit Haemek, Israel. Insulin RIA was obtained from Sorin Biomedica (Saluggia, Italy) and the rat insulin standards from LINCO Research, Inc., (St. Louis, Mo., USA). The rabbit anti-GLUT-2 antibody was purchased from Chemicon, Inc., (Temecula, Ca., USA). A major band on membrane preparation was recognized at approximately 53–61 kDa. Peroxidase-labeled anti-rabbit-IgG antibody from Jackson Laboratories, Inc. (West Grove, Pennsylvania, USA) was used as a second antibody. The ECL detection system was purchased from Amersham (Braunschweig, Germany) and the protein molecular weight standards from GIBCO Life Technologies (Vienna, Austria).

### Cell Culture and Selection Procedure

The RINm cells, which have been previously described in detail,[3] were kindly provided by M. Walker (Weizmann Institute of Science, Rehovot, Israel). Cells were cultured in RPMI 1640 medium supplemented with 10% FCS, 2 mmol/l L-glutamine, 100 IU penicillin and 100 μg/ml streptomycin. Cells were grown in plastic tissue culture flasks at 37°C in 95% humidified air with 5% $CO_2$. Cells free of micoplasma contamination were used in all experiments. Resistant cell subpopulations were obtained following repeated exposure of parental RINm cells to STZ or AL. Briefly, cells were grown to near confluence in culture flasks and then incubated for 1 hr in glucose-free Krebs-Ringer bicarbonate buffer, supplemented with 10 mM HEPES, bovine albumin 0.1% (KRB), containing 10 mM STZ. Following this procedure, approximately 10% of the cells remain attached to the flask wall. When these cells reached semiconfluence upon culture, a second STZ treatment was performed as described above. A similar procedure was used with 20 mM of AL in order to obtain an AL-resistant cell subpopulation. In parallel, the control parental RINm cells were incubated twice with KRB alone. In this study we used only cells from passages 14–22.

### Determination of Cell Resistance to STZ, AL and Hydrogen Peroxide

After exposure of the cells to various concentrations of toxins, cell resistance was evaluated by measurments of cell viability and proliferation. Cell viability was determined by the MTT colorimetric assay, which reflects mitochondrial oxidative processes of living cells.[14] Briefly, cells were cultured for 3 days in 24-well plates ($2 \times 10^5$ cells/ml/well) and then exposed for 2 hr to a glucose-free KRB solution containing various concentrations of STZ (0–10 mmol/l) or AL(0–10 mmol/l). The STZ solution was replaced by KRB medium supplemented with 0.5 mg/ml MTT for a period of 2 hr. The MTT-containing medium was then removed, and the cells were exposed to 2 ml isopropanol. The reduction of tetrazolium salt to formazan was quantified by measuring optical density with a spectrophotometer at 540 nm. Cell proliferative activity was determined by [$^3$H] thymidine incorporation assay. Cells ($0.5 \times 10^5$/200 µl/well) were cultured for 24 hr in 96-well microplates and then exposed for 2 hr to various concentrations of STZ or AL and pulsed for 18 hr with 1 µCi/well of [$^3$H] thymidine. The cells were harvested by the Micromate 196 instrument (Packard, Switzerland), and the level of incorporated [$^3$H] thymidine was estimated by counting scintillation radioactivity. Results were expressed as percent of MTT reduction or thymidine incorporation in the absence of toxins. In order to estimate the toxic effect of hydrogen peroxide which is a GLUT-2 independent compound, the cells were exposed to 50 mol/l $H_2O_2$ for 20 and 60 min in Dulbecco's phosphate-buffered saline, pH 7.4 at 37°C. The cells were then washed and cultivated in culture medium for 18 hr with 1 µCi/well of [$^3$H] thymidine.

### Effect of 3-OMG on STZ Toxicity

GLUT-2 involvement in beta-cell sensitivity to toxins was indirectly estimated by preincubation of parental RINm, RINmS and RINmA cells with 20 mM of non-metabolizable 3-OMG for 30 min prior to STZ and AL exposure. The expected competitive inhibitory effect of 3-OMG on cytotoxicity was determined by [$^3$H] thymidine incorporation assay.

### Western Blot Analysis of GLUT-2

Membrane GLUT-2 pellets of parental and resistant cells were resuspended in a sample buffer as previously described.[15] Sixty µg of total membrane protein/well were resolved in 10% SDS-polyacrylamide gel, with the vertical mini-gel system MGV-100 (C.B. S. Scientific Co., USA). The protein was then electroblotted to Hybond-ECL nitrocellulose membrane (Amersham) at 22 V for 1.20 h with the semi-dry blotter EBU-4000 (C.B.S. Scientific Co.). The membrane was washed for 30 min in PBS with 0.1% Tween-20 (PBS-T) and then blocked overnight in PBS-T with 5% non-fat dry milk at 4°C. Following five 10-min washing steps in PBS-T, the membrane was incubated with a rabbit anti-GLUT-2 antibody (1 : 5000 dilution), washed in PBS-T, the membrane was incubated for 1 h with peroxidase-labeled anti-rabbit -IgG antibody (1 : 10 000 dilution) in 5% milk PBS-T. The specific protein bands were visualized by chemiluminescence using ECL detection system.

### Determination of Intracellular Insulin Content and Secretion

Following trypsinization, the cells were harvested, counted, and their intracellular insulin content was determined by radioimmunoassay (RIA) after sonication for 15 seconds and acid ethanol extraction of insulin. The level of insulin secreted in to the culture medium (11 mM of glucose) was determined by RIA.

### Determination of Cellular Chromosome Number

Growing cells were treated with colchicine (0.25 μg/ml medium) for 1.5 hr at 37°C. Following trypsin-EDTA treatment, detached cells were centrifuged, pellet prepared and fixed by cold methanol/acetic acid (3 : 1, vol./vol.). Chromosomes were spread by dropping the suspension onto ice-cold microscope slides and allowed to air dry. Preparations were stained with freshly prepared Giemsa stain. The cell chromosome number was determined by counting metaphases of 1000 cell of each type.

### Statistical Analysis

Analysis of variance (ANOVA) was utilized for evaluation of the statistical significance of differences between groups. The results are presented as mean values ± SEM of independent, repeated experiments. Experiments were done in triplicate; $p$ values $< 0.05$ were considered significant.

### RESULTS

### Cell Resistance to Various Toxins

Higher cell viability following toxin treatment was found in RINmS and RINmA than parental RINm cells using both the MTT (Fig. 1) and [$^3$H]thymidine incorporation assay (Fig. 2). However, we did not find any differences in sensitivity of parental and toxin selected cells to hydrogen peroxide (Fig. 3). Because the [$^3$H] thymidine incorporation assay was significantly more sensitive than the MTT method, we used the radioactive assay in all experiments for determining STZ and AL toxicity. As sensitivity to STZ and AL depends on their penetration to the cell and 3-OMG may reduce toxin's penetration by blocking GLUT2,[7,8] we studied the competitive effect of 3-OMG on the cell reaction to toxin exposure.

### Effect of 3-OMG on STZ and AL Toxicity

Incubation of the cells with the unmetabolized sugar, 3-OMG (20 mM) prior to toxin treatment, rendered the parental RINm cells more resistant to STZ (10 mM) and AL (10 mM) action, as expressed by increased level of [$^3$H] thymidine incorporation. In contrast, RINmS and RINmA sensitivity to STZ (10 mM) and AL (10 mM) was not affected by this procedure (Fig. 4). These data suggest that GLUT-2 level plays a role in toxin accessibility to the cell.

### Western Blot Analysis of GLUT-2

Using rabbit anti-GLUT-2 polyclonal antibody, GLUT-2 protein was detected at 60 kDa in both parental cells and toxin-selected cell subpopulations. However, the level of GLUT-2 expression was lower in RINmS and RINmA cells than that found in the parental RINm cells (Fig. 5). The possibility that poor GLUT-2 expression in the selected cells would reduce the cell capacity to produce insulin was then investigated.

### Intracellular Insulin Content and Secretion

Interestingly, the intracellular insulin concentration of RINmA and RINmS cells was

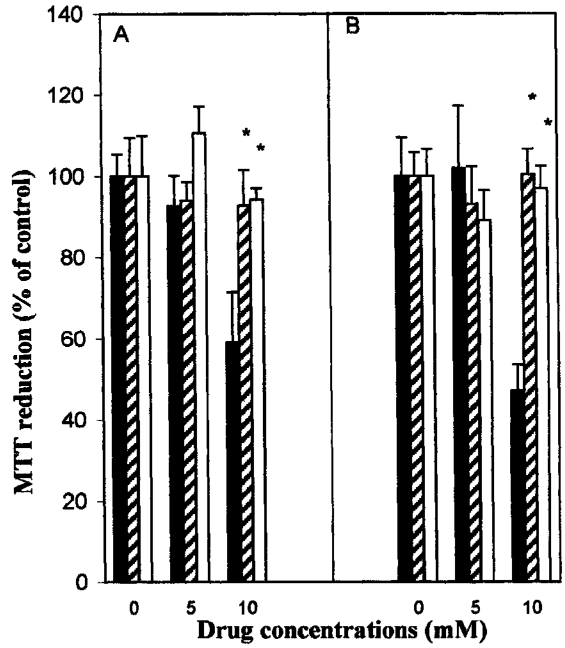

FIGURE 1 Viability (estimated by MTT assay) of RINm (black bar), RINmS (hatched bar) and RINmA (open bar) cells exposed to STZ (A) and AL (B). Results are expressed as percent of MTT reduction compared to control values (0 mM). Data are given as mean values ± SEM ($n = 3$). $^*p < 0.05$ compared to RINm cells.

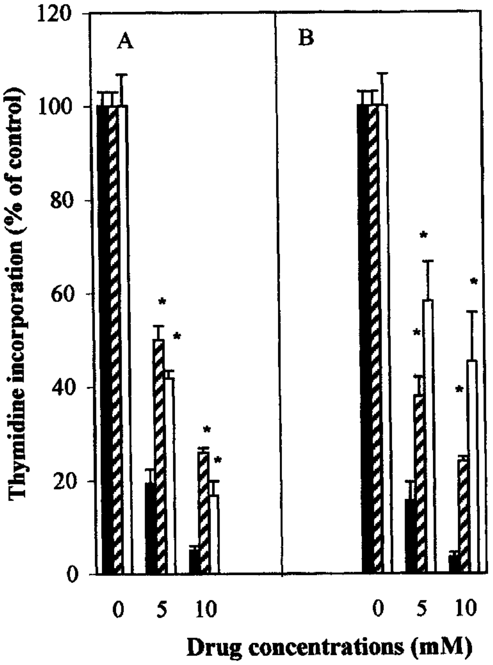

FIGURE 2 [$^3$H] thymidine incorporation in RINm (black bar), RINmS (hatched bar) and RINmA (open bar) cells exposured to STZ (A) and AL (B). Results are expressed as percent of [$^3$H] thymidine incorporation as compared to control (0 mM). Data are given as mean values ± SEM ($n = 3$). $^*p < 0.05$ compared to RINm cells.

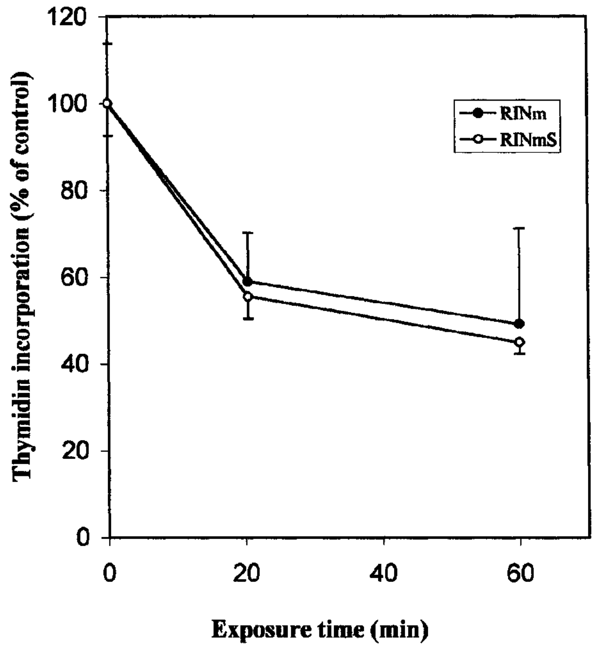

FIGURE 3 [$^3$H] thymidine incorporation in RINm (black circle) and RINmS (open circle) cells exposured to 50 μmol/l $H_2O_2$. Results are expressed as percent of [$^3$H] thymidine incorporation as compared to control (0 μmol/l). Data are given as mean values ± SEM ($n = 3$).

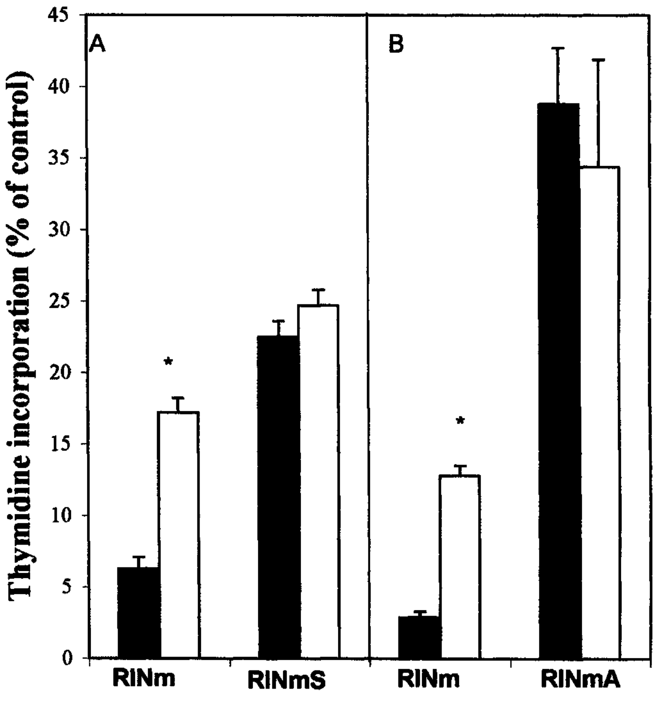

FIGURE 4 Effect of 3-OMG on STZ (A) and AL (B) cytotoxicity as measured in the thymidine incorporation assay. Prior to STZ or AL treatment (10 mM), cells were incubated with 3-OMG (20 mM) for 0.5 hr. Black bars – STZ or AL treatment alone, open bars – 3-OMG + drug. Values represent means ± SE for 3 independent assays. *$p < 0.05$ compared to RINm cells incubated with STZ or AL alone.

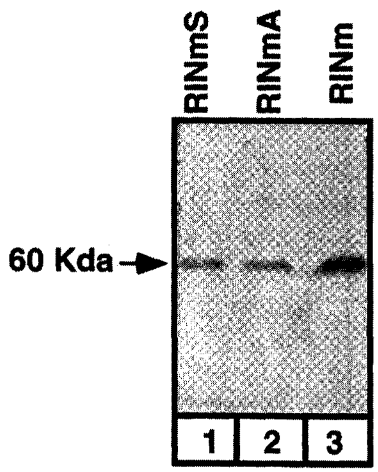

FIGURE 5 Western blot analysis of GLUT-2 in parental RINm and toxin selected RINmS and RINmA cells. GLUT-2 protein was detected by immunoblotting as described in MATERIALS AND METHODS, using 60 µg protein per lane. The GLUT-2 protein is demonstrated by a distinct band (~60 Kda). The blot shown is representative of 3 independent experiments.

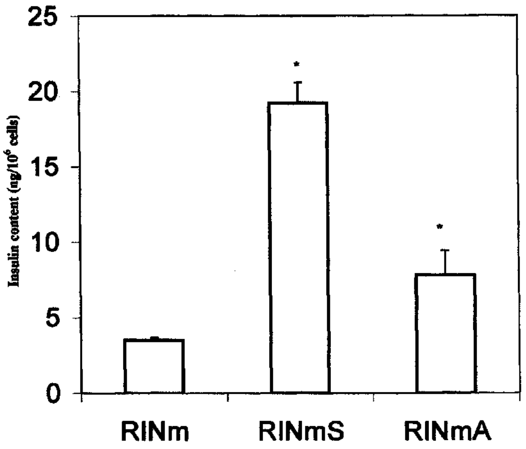

FIGURE 6 Insulin content in RINm, RINmS and RINmA cells. Values represent means ± SEM of 6 independent assays. *$p < 0.05$ compared to RINm cells.

significantly higher (2.2 and 5.5-fold, respectively) than in the parental RINm cells under identical conditions (Fig. 6), suggesting intact cell function. In addition, insulin released by RINmA and RINmS (16.8 ± 1.2 and 17.6 ± 3.0 ng/24 h/$10^6$ cells, respectively) into the culture medium (11 mM of glucose) was approximately 2.5-times higher of that secreted by the parental RINm cells (6.46 ± 0.7 ng/24 h/$10^6$ cells).

### Chromosome Number

Cytogenetic analysis of the chromosome number indicated that the majority of parental as well as STZ and AL selected cells, was composed of hypodiploid cells with a chromosome number of 38 to 41 ($2n = 42$). However, toxin selected cells had a higher frequency of polyploid cells (1.5–2.2%) than parental RINm cells (0.7%).

### DISCUSSION

The defense properties of beta-cells are believed to be crucial to their long-term survival after transplantation. In the present study, we demonstrated using two different methods, that selection of cell subpopulations with improved defense mechanisms can be obtained upon repeated exposure of tumoral insulin-producing beta-cells to high doses of STZ and AL. The STZ resistance obtained using this procedure, may be explained by the direct relation between the cytotoxic effect of this compound and the level of GLUT-2 expression in the beta-cells.[7] Such a relationship suggests that GLUT-2 involvement in STZ cytotoxicity is associated with the specific recognition of STZ as a transportable substrate. Interestingly, no difference in sensitivity to oxygen free radical donor, such as hydrogen peroxide, was found between toxin selected and parental cells, suggesting a different pathway of cytotoxic activity then STZ and AL. The toxin-based selection protected the surviving cells from STZ as well as from AL. Our observation that GLUT-2 mediates not only STZ but also AL uptake into the beta-cells is supported in the literature.[8] GLUT-2 is known to be the major glucose transporter isoform expressed in rodent beta-cells.[16] Therefore, it is possible that the cell resistance obtained in RINmS and RINmA cells was due to the selection of surviving cells with a reduced expression of GLUT-2. Indeed, Western blot analysis showed that the toxin-resistant RIN cells when compared with parental ones expressed a lower level of GLUT-2 protein. Moreover, a competitive inhibition of STZ and AL cytotoxicity by the unmetabolized 3-OMG could be demonstrated only in parental RINm cells. 3-OMG binds to and is transported by a pancreatic beta-cell membrane glucose transporter, but it is not metabolized in the beta-cell itself.[5, 8] Our results are in agreement with the reported deficiency of GLUT-2 expression in pancreatic islets isolated from multiple low-dose STZ-treated mice.[17] As 3-OMG did not confer complete cell protection from STZ action, we assume that additional factors, such as free radical scavenging enzymes and repair genes, are involved in cell defense mechanisms. Indeed, our data indicate that the cytotoxic effect of $H_2O_2$ is not influenced by the different GLUT-2 expression of the two cell population in the study, and a decrease in GLUT-2 expression, is not thus expected to protect beta cells from immune-mediated cell death. The finding that the level of cellular insulin content and insulin secretion in RINmS and RINmA was higher than in the parental RINm cells, was unexpected, particularly due to the finding of lower GLUT-2 in these subpopulations. Such insulin enrichment of toxin-treated cells could have resulted from the transition of this cell population to a more differentiated stage. This suggestion is in agreement with a recent demonstration differentiating PDX-1 positive beta-stem cells into insulin-producing cells following STZ injury. STZ was found to induce expression of PDX-1 transcription factor in a population of somatostatin-producing cells, which differentiated into insulin-producing cells.[10] As RINm cell lines are known to be composed not only of insulin, but also of somatostatin-producing cells,[3] this latter cell population could act as a multipotential source for toxin-inducible, insulin-producing cells. Transition of cells from a one stage to another following toxin injury was also demonstrated by the increased rate of cell polyploidization, a phenomenon previously described in pancreatic beta-cells of diabetic animals.[18]

Our data suggest that STZ and AL treatment can be used as a method for selection of toxin-resistant subpopulations of RIN cells. Although selected resistant cells obtained by this method express a low level of GLUT-2, their functional capacity as reflected by intracellular insulin content and insulin secretion does not seem to be impaired. Further investigation is needed in order to elucidate the mechanisms leading to this phenomenon and to evaluate its utility in non-tumor-derived insulin-producing cells.

Such research would allow future transplantation of highly protected and functional insulin-producing cells from various sources.

### Acknowledgment

This work was partially supported by grant #6774195 of the Israeli Ministry of Science and Art.

### References

[1] Newgard, C. B. (1994). Cellular engineering and gene therapy strategies for insulin replacement in diabetes, *Diabetes*, **43**, 341–350.

[2] Efrat, S. (1998). Cell-based therapy for insulin-dependent diabetes mellitus, *Eur. J. Endocrinol.*, **138**, 129–133.

[3] Gazdar, A. F., Chick, W. L., Oie, H. K., Sims, H. L., King, D. L., Weir, G. C. and Lauris, V. (1980). Continuous, clonal, insulin- and somatostatin-secreting cell lines established from a transplantable rat islet cell tumor, *Proc. Natl. Acad. Sci. USA*, **77**, 3519–3523.

[4] Okamoto, H. (1996). Okamoto model for B-cell damage: recent advances, In: *Lessons from animal diabetes*, edited by Shafrir, E. pp. 97–112, Boston, Birkhauser.

[5] Lenzen, S. and Panten, U. (1988). Alloxan: history and mechanism of action, *Diabetologia*, **31**, 337–342.

[6] Weiss, R. B. (1982). Streptozotocin: a review of its pharmacology, efficacy, and toxicity, *Cancer Treat Rep.*, **66**, 427–438.

[7] Schnedl, W. J., Ferber, S., Johnson, J. H. and Newgard, C. B. (1994). STZ transport and cytotoxicity. Specific enhancement in GLUT-2-expressing cells, *Diabetes*, **43**, 1326–1333.

[8] Munday, R., Ludwig, K. and Lenzen, S. (1993). The relationship between the physicochemical properties and the biological effects of alloxan and several N-alkyl substituted alloxan derivatives, *J. Endocrinol.*, **139**, 153–163.

[9] Tiege, M., Lortz, S., Drinkgern, J. and Lenzen, S. (1997). Relation between antioxidant enzyme gene expression and antioxidative defense status of insulin-producing cells, *Diabetes*, **46**, 1733–1742.

[10] Fernandes, A., King, L. C., Guz, Y., Stein, R., Wright, C. V. E. and Teitelman, G. (1997). Differentiation of new insulin-producing cells is induced by injury in adult pancreatic islets, *Endocrinology*, **138**, 1750–1762.

[11] Eizirik, D. L., Pipeleers, D. G., Ling, Z., Welsh, N., Hellerstrom, C. and Andersson, A. (1994). Major species differences between humans and rodents in the susceptibility to pancreatic beta-cell injury, *Proc. Natl. Acad. Sci. USA*, **91**, 9253–9256.

[12] De Vos, A., Heimberg, H., Quartier, E., Huypens, P., Bouwens, L., Pipeleers, D. and Schuit, F. (1995). Human and rat beta cells differ in glucose transporter but not in glucokinase gene expression, *J. Clin. Invest.*, **96**, 2489–2495.

[13] Pipeleers, D., Kiekens, R., Ling, Z., Wilikens, A. and Schuit, F. (1994). Physiologic relevance of heterogeneity in the pancreatic beta-cell population, *Diabetologia*, **37** (Suppl. 2), S57–S64.

[14] Janjic, D. and Wollheim, C. B. (1992). Islet cell metabolism is reflected by the MTT (tetrazolium) colorimetric assay, *Diabetologia*, **35**, 482–485.

[15] McClenaghan, N. H., Barnet, C. R., Ah-Sing, E., Abdel-Wahab, Y. H. A., O'Harte, F. P. M., Yoon, T. W., Swanston-Flatt, S. K. and Flatt, P. R. (1996). Characterization of a novel glucose-responsive insulin-secreting cell line, BRIN-BD11, produced by electrofusion, *Diabetes*, **45**, 1132–1140.

[16] Thorens, B., Sarkar, H. K., Kaback, H. R. and Lodish, H. F. (1988). Cloning and functional expression in bacteria of a novel glucose transporter present in liver, intestine, kidney, and beta-pancreatic islet cells, *Cell*, **55**, 281–290.

[17] Wang, Z. and Gleichmann, H. (1998). GLUT-2 in pancreatic islets: crucial target molecule in diabetes induced with multiple low doses of streptozotocin in mice, *Diabetes*, **47**, 50–56.

[18] White, J. W., Swartz, F. J. and Swartz, A. F. (1985). Excess glucose intake induces accelerated beta-cell polyploidization in normal mice: a possible deleterious effect, *J. Nutr.*, **115**, 271–278.

